# Controlling Resonator Nonlinearities and Modes through Geometry Optimization

**DOI:** 10.3390/mi12111381

**Published:** 2021-11-10

**Authors:** Amal Z. Hajjaj, Nizar Jaber

**Affiliations:** 1 Wolfson School of Mechanical, Electrical and Manufacturing Engineering, Loughborough LE11 3TU, UK; 2 Mechanical Engineering Department and the Centre for Communications Systems and Sensing, King Fahd University of Petroleum and Minerals, Dhahran 31261, Saudi Arabia; nizar.jaber@kfupm.edu.sa

**Keywords:** MEMS resonators, nonlinearity tailoring, geometry optimization

## Abstract

Controlling the nonlinearities of MEMS resonators is critical for their successful implementation in a wide range of sensing, signal conditioning, and filtering applications. Here, we utilize a passive technique based on geometry optimization to control the nonlinearities and the dynamical response of MEMS resonators. Also, we explored active technique i.e., tuning the axial stress of the resonator. To achieve this, we propose a new hybrid shape combining a straight and initially curved microbeam. The Galerkin method is employed to solve the beam equation and study the effect of the different design parameters on the ratios of the frequencies and the nonlinearities of the structure. We show by adequately selecting the parameters of the structure; we can realize systems with strong quadratic or cubic effective nonlinearities. Also, we investigate the resonator shape effect on symmetry breaking and study different linear coupling phenomena: crossing, veering, and mode hybridization. We demonstrate the possibility of tuning the frequencies of the different modes of vibrations to achieve commensurate ratios necessary for activating internal resonance. The proposed method is simple in principle, easy to fabricate, and offers a wide range of controllability on the sensor nonlinearities and response.

## 1. Introduction

The Resonant microstructures have demonstrated their potential in sensing [1,2,3], filtering [4,5], logic devices [6,7], energy harvesting [8,9], and signal processing and also timing applications [10,11]. Traditionally, many of these applications require operating the resonator in the linear regime. However, as the dimensions of the resonator are shrunk, their response becomes nonlinear due to the size and surface effects and quantum forces. These nonlinearities can be attributed to the midplane stretching effect, material nonlinearity, and the nonlinear nature of the actuation force [12,13]. Although at the nano and micro-scale these nonlinearities are inherently presented, one can introduce nonlinearities at the macro scale by using materials that have a nonlinear stress-strain relation or imposing certain geometrical constraints or flexible boundary conditions. Usually, nonlinear behavior is considered undesirable and might lead to the failure of the device. Yet, advances in the field of nonlinear dynamics led to the creation of a new class of devices that make use of the nonlinear phenomena in applications such as imaging [14,15,16], ultra-sensitive mass sensor [3], energy harvesting [17], filtering [18] and frequency divider [19].

Recently, researchers investigated different methods for tailoring nonlinearities to achieve targeted responses. These methods can be classified into two categories: active and passive techniques. In active techniques, the nonlinearities can be controlled over the lifetime of the device using electrostatic [20,21], electrothermal [22,23], and electromagnetic actuation [24]. Active methods are implemented to overcome fabrication imperfection and impedance mismatching, but they suffer from the need for additional circuits, high power consumption, and increases the device size and cost. On the other hand, passive techniques rely on optimizing the device geometry and selecting materials with desired inherent nonlinearities. Cho et al. [25] showed by controlling the orientation of the nanotube and introducing a small nanoscale attachment on the structure a strong quadratic nonlinearity can be induced that shifts the resonance frequency from the hardening to softening resonance. Alcheikh et al. [26,27] investigated the dynamics of stepped and V-shaped microbeams and highlighted the possibility of tuning the natural frequencies and nonlinearities by selecting different geometrical parameters. In [28], gradient-based method has been employed to optimize the device shape and achieve a targeted nonlinear behavior. This method resulted in beams with nonuniform profile that can have wide range of duffing nonlinearities values [29]. A procedure to design different structures with commensurable natural frequencies to activate internal resonance is developed in [30]. Indeed, internal resonance (autoparametric resonance) represents a mechanism for nonlinear energy transfer from the targeted vibration mode to a different vibration mode. For the activation of this nonlinear modal coupling, the two involved modes need to have an integer ratio (commensurate ratio). Shoshani et al. [31] demonstrated the possibility of controlling the nonlinearity by coupling the primary resonator to a secondary resonator.

Tailoring the resonator nonlinearity affects the frequency tuning of the structure, leading to mode coupling of different vibration modes. Recently, linear and nonlinear mode coupling of microresonators has increasingly attracted researcher attention showing their potential exploitation for various applications [32,33,34,35,36]. Also, nonlinear mode coupling was investigated for gyroscopic ring resonators electrostatically actuated [37,38]. Moreover, initially curved resonators are among the widely exploited structures in the literature due to their remarkable ability to activate linear and nonlinear mode coupling [18,39,40,41]. The high tunability of the modes as function of the design parameters (axial force, initial rise, curvature shape, and thickness) is considered a key feature of these structures [22,23]. Hajjaj et al. [23] demonstrated different tunability scenarios as changing the beam geometry and tuning its axial load. Coupling among the different modes of vibration can be observed as varying the axial load. The coupling can be linear in case of veering (avoided-crossing) and nonlinear in case of internal resonance. 

On the one hand, as two frequencies approach each other while tuning a control parameter, they either crossover; mainly occurs among two frequencies of symmetric and antisymmetric modes; or veer from each other; occurring among symmetric and/or antisymmetric modes (veering phenomena). In the veering zone, the mode shapes of the interacting modes get affected, which is identified as mode hybridization. In the microscale, the veering phenomenon is mostly associated with the mode-localization phenomenon occurring in weakly coupled systems [42,43]. Recently, veering for internally coupled modes in MEMS resonators with highly tunable stiffness is reported for arc and V-shaped resonators [18,23,27]. More recently, Hajjaj and co-authors [18,23,44,45] investigated the veering phenomenon experimentally and theoretically in the case of MEMS arc resonators and demonstrated its high capabilities in sensing and filtering applications. 

Tuning the axial load of initially curved resonators can be used to control the ratio between different vibration modes. An integer ratio is a must condition for nonlinear energy transfer among different modes (internal resonance phenomenon). Different types of internal resonances were investigated in-depth numerically and experimentally as tuning the ratio between different modes (1:1, 2:1, 3:1, and 4:1) [44,45]. Internal resonance showed a high potential in several applications, such as sensing [3,46,47], synchronization [48], and communication [10,45]. 

**Figure 1:**
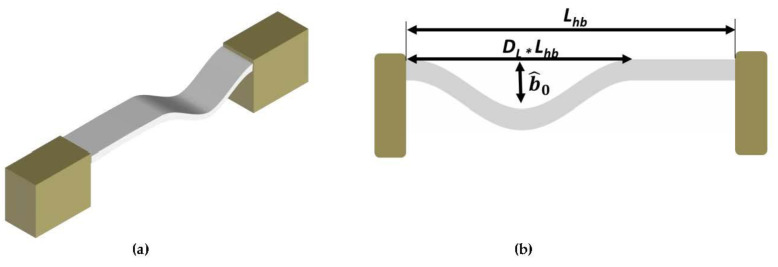
(**a**,**b**) 3D and 2D schematic of the in-plane clamped-clamped- hybrid beam resonator, respectively.

In this work, we explore a newly hybrid-shape microbeam composed of an arch (combining quadratic; originated from curvature; and cubic; originated from midplane stretching; nonlinearities) and a straight beam (dominated by cubic nonlinearity originated from midplane stretching), Figure 1, to induce a geometrical nonlinearity that can be tuned to achieve the targeted nonlinear phenomenon. The presented concept is simple in principle, scalable (macro, micro, and nano scales), and can be employed to achieve a wide range of targeted responses.

## 2. Materials and Methods

We propose a new structure that combines the straight and buckled shape in the same design to take advantage of both configurations on the resonator behavior. We introduce a buckled shape dome of length *D_L_* × *L_hb_* in the beam configuration, Figure 1. The initial shape ${\hat{w}}_{hb,0}\left(\hat{x}\right)$ of the clamped-clamped hybrid beam, of length *L_hb_*, is described by Equation (1).

(1) $${\hat{w}}_{hb,0}\left(\hat{x}\right)=-\frac{{\hat{b}}_{0}}{2}\left(1-\cos\left(2\pi \frac{\hat{x}}{D_{L}}\right)\right)U\left[D_{L}\times L_{hb}-\hat{x}\right]$$

Here $\hat{x}$ denotes the position along the beam length, and ${\hat{b}}_{0}$ represents the dome midpoint rise. $U\left[\hat{x}\right]$ denotes the unit-step function. The cross-sectional area of the hybrid beam is assumed to be rectangular $A=b_{hb}h_{hb}$ with a moment of inertia, $I=b_{hb}h_{hb}^{3}/12$ where *b_hb_* and *h_hb_* denote the width and the thickness, respectively. 

As a case study, we consider a microbeam made from silicon and with dimensions given in Table 1. 

The equation of motion governing the transverse vibration of the hybrid beam is given by [23]:

(2) $$\rho b_{hb}h_{hb}\frac{\partial^{2}{\hat{w}}_{hb}}{\partial {\hat{t}}^{2}}+EI\frac{\partial^{4}{\hat{w}}_{hb}}{\partial {\hat{x}}^{4}}+\hat{c}\frac{\partial {\hat{w}}_{hb}}{\partial \hat{t}}=\left\{\left(\frac{\partial^{2}{\hat{w}}_{hb}}{\partial {\hat{x}}^{2}}+\frac{d^{2}{\hat{w}}_{hb,0}}{d{\hat{x}}^{2}}\right)\times \left[\hat{N}+\;\frac{EI}{L_{hb}}\int_{0}^{L_{hb}}\left({\left(\frac{\partial {\hat{w}}_{hb}}{\partial \hat{x}}\right)}^{2}+2\frac{\partial {\hat{w}}_{hb}}{\partial \hat{x}}\frac{d{\hat{w}}_{hb,0}}{d\hat{x}}\right)d\hat{x}\right]\right\}$$

The resonating beam is subjected to the following clamped-clamped boundary conditions:

(3) $${\hat{w}}_{hb}\left(0,\hat{t}\right)={\hat{w}}_{hb}\left(L_{hb},\hat{t}\right)=0;\;\mathrm{and}\;{\left.\frac{\partial {\hat{w}}_{hb}}{\partial \hat{x}}\right|}_{\left(0,\hat{t}\right)}={\left.\frac{\partial {\hat{w}}_{hb}}{\partial \hat{x}}\right|}_{\left(L_{hb},\hat{t}\right)}=0$$

Equations (2) and (3) are normalized by using the following nondimensional variables:

(4) $$w_{hb}=\frac{{\hat{w}}_{hb}}{r}\;;\;x=\frac{\hat{x}}{r}\;;\;t=\frac{\hat{t}}{T_{S}};\;w_{hb,0}=\frac{{\hat{w}}_{hb,0}}{r};\;\mathrm{and}\;b_{0}=\frac{{\hat{b}}_{0}}{r}$$

where r=IA denotes the gyration radius of the cross-section, and $T_{s}=\sqrt{\frac{\rho b_{hb}h_{hb}L_{hb}^{4}}{EI}}$ is the timescale. Substituting Equation (4) into Equations (2) and (3), we obtain the non-dimensionalized equation of motion and associated boundary conditions:

(5) $$\frac{\partial^{2}w_{hb}}{\partial t^{2}}+\frac{\partial^{4}w_{hb}}{\partial x^{4}}+c\frac{\partial w_{hb}}{\partial t}=\left(\left(\frac{\partial^{2}w_{hb}}{\partial x^{2}}+\frac{d^{2}w_{hb,0}}{dx^{2}}\right)\left[N_{non}+\frac{1}{2}\int_{0}^{1}\left[{\frac{\partial w_{hb}}{\partial x}}^{2}+2\frac{\partial w_{hb}}{\partial x}\frac{dw_{hb,0}}{dx}\right]dx\right]\right)$$

(6) $$w_{hb}\left(0,t\right)=w_{hb}\left(1,t\right)=0;\;\mathrm{and}\;{\left.\frac{\partial w_{hb}}{\partial x}\right|}_{\left(0,t\right)}={\left.\frac{\partial w_{hb}}{\partial x}\right|}_{\left(1,t\right)}=0$$

The nondimensional parameters appearing in Equation (5) are defined as follow:

(7) $$N_{non}=\frac{L_{hb}^{2}}{EI}\hat{N};c=\frac{L_{hb}^{4}}{EIT_{s}}\hat{c}\;$$

To study the variation of the natural frequencies of the hybrid beam due to the alteration of the axial load, *N_non_*, we start by linearizing the equation of motion describing the small dynamic behavior of the arch beam around the new static configuration governed by Equation (9) [23].

(8) $$\frac{d^{4}w_{hb,s}}{dx^{4}}=\left(\left(\frac{d^{2}w_{hb,s}}{dx^{2}}+\frac{d^{2}w_{hb,0}}{dx^{2}}\right)\left[N_{non}+\frac{1}{2}\int_{0}^{1}\left[{\left(\frac{dw_{hb,s}}{dx}\right)}^{2}+2\frac{dw_{hb,s}}{dx}\frac{dw_{hb,0}}{dx}\right]dx\right]\right)$$

with the associated boundary conditions

(9) $$w_{hb,s}\left(0\right)=w_{hb,s}\left(1\right)=0\;\mathrm{and}\;{\left.\frac{dw_{hb,s}}{dx}\right|}_{x=0}={\left.\frac{dw_{hb,s}}{dx}\right|}_{x=1}=0$$

Next, after dropping the damping terms from the equation of motion, Equation (5), the total deflection is assumed to be equal to the sum of the static configuration, *w_hb,s_(x)*, and a small dynamic deflection of the hybrid beam, *w_hb,d_(x,t)*, around *w_hb,s_(x)*. The outcome equation becomes

(10) $$\begin{array}{cc} \frac{\partial^{2}w_{hb,d}}{\partial t^{2}}+\frac{\partial^{4}w_{hb,d}}{\partial x^{4}} & \\ & =\left[N_{non}+\frac{1}{2}\int_{0}^{1}\left[{\left(\frac{dw_{hb,s}}{dx}\right)}^{2}+2\frac{dw_{hb,s}}{dx}\frac{dw_{hb,0}}{dx}\right]dx\right]\frac{\partial^{2}w_{hb,d}}{\partial x^{2}}\\ & +\int_{0}^{1}\left[\left(\frac{dw_{hb,s}}{dx}+\frac{dw_{hb,0}}{dx}\right)\frac{\partial w_{hb,d}}{\partial x}\right]dx\left(\frac{d^{2}w_{hb,s}}{dx^{2}}+\frac{d^{2}w_{hb,0}}{dx^{2}}\right) \end{array}$$

with the associated boundary conditions.

(11) $$w_{hb,d}\left(0,t\right)=w_{hb,d}\left(1,t\right)=0\;\mathrm{and}\;{\left.\frac{\partial w_{hb,d}}{\partial x}\right|}_{x=0,t}={\left.\frac{\partial w_{hb,d}}{\partial x}\right|}_{x=1,t}=0$$

The eigenvalue problem is solved using the Galerkin discretization. Toward this, we let

(12) $$w_{hb,d}\left(x,t\right)=\sum_{i=1}^{n}q_{i}\left(t\right)\varphi_{i}\left(x\right)$$

where *q_i_(t)* (*i =* 1, 2..., *n*) denotes the nondimensional modal coordinates and *φ_i_(x)* (*i =* 1, 2..., *n*) denotes the mode shape of the unforced and undamped clamped-clamped standard arch beam [16]. 

Next, we substitute Equation (12) into Equation (10), multiply the outcome by the mode shape *φ_j_*, and integrate over the beam domain, which yields the following equation:

(13) $${\ddot{q}}_{j}=-\int_{0}^{1}\varphi_{j}\left(\sum_{i=1}^{n}q_{i}\varphi_{i}{}^{\left(iv\right)}\right)dx+\left[N_{non}+\frac{1}{2}\int_{0}^{1}\left[{\left(\frac{dw_{hb,s}}{dx}\right)}^{2}+2\frac{dw_{hb,s}}{dx}\frac{dw_{hb,0}}{dx}\right]dx\right]\int_{0}^{1}\varphi_{j}\left(\sum_{i=1}^{n}q_{i}\varphi_{i}^{\prime \prime }\right)dx\;+\int_{0}^{1}\left[\left(\frac{dw_{hb,s}}{dx}+\frac{dw_{hb,0}}{dx}\right)\sum_{i=1}^{n}q_{i}{\varphi_{i}}^{\prime }\right]dx\int_{0}^{1}\varphi_{j}\left(\frac{d^{2}w_{hb,s}}{dx^{2}}+\frac{d^{2}w_{hb,0}}{dx^{2}}\right)dx$$

Using 5 modes, the static deflection and then the Jacobian of the five obtained equations are computed for each *N_non_*. Then, we calculate the resonators’ natural frequencies, at constant *N_non_*, by taking these eigenvalues’ square root. A convergence analysis was conducted in previous works to determine the number of exact modes needed to simulate the curved resonator behavior [23,49].

## 3. Results

To explore the potential of the proposed hybrid design in controlling the nonlinearities and the microbeam frequencies, we solve the beam equation for a different set of dome length, initial rise, and axial stress. The geometric and mechanical parameters of the hybrid beam used in the simulations are given in Table 1. To calculate the static deflection and eigenvalue, we used five modes of the arch configuration needed to reach convergence.

**Figure 2:**
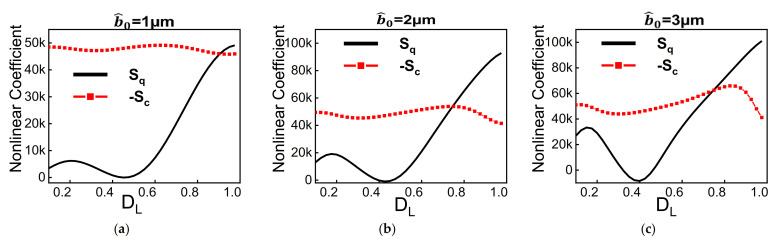

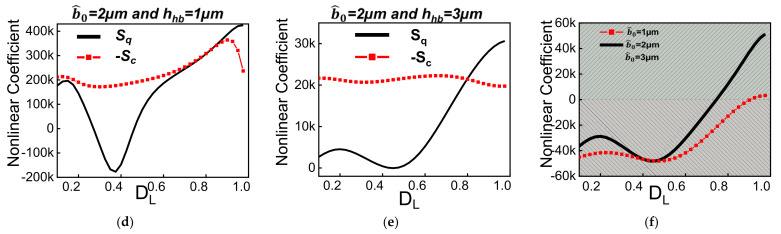
Variation of cubic (dashed) and quadratic (solid) nonlinearity coefficients of the proposed hybrid curved beam with dome length. (**a**) ${\hat{b}}_{0}$ *=* 1 µm and *h =* 2 µm (**b**) ${\hat{b}}_{0}$ = 2 µm and *h_hb_* = 2 µm (**c**) ${\hat{b}}_{0}$ = 3 µm and *h_hb_* = 2 µm (**d**) ${\hat{b}}_{0}$ = 2 µm and *h_hb_* = 1 µm (**e**) ${\hat{b}}_{0}$ = 2 µm and *h_hb_* = 3 µm. (**f**) the total effective nonlinearity for *h_hb_* = 2 µm at different initial rises. The *D_L_* axis starts from 0.1.

Firstly, to analyze the effect of the different geometry parameters on the nonlinearity’s type and magnitude, we compute the quadratic and cubic nonlinearity coefficients of the proposed structure (the derivation is presented in the Appendix A). As shown in Figure 2a–c, the quadratic nonlinearity is more sensitive to the dome length and changes rapidly for a fixed initial rise and thickness. Also, increasing the initial rise expands the quadratic nonlinearity range with minimal effect on the cubic nonlinearity. Figure 2d,e show that increasing the thickness from 1 µm to 3 µm, for an initial rise equals to 2 µm, reduces the cubic nonlinearity by order of magnitude. As revealed in Figure 2f, designing a microstructure with strong quadratic, cubic, or even zero effective nonlinearity can be easily achieved by appropriately selecting the dome length, beam thickness, and initial rise. One should mention that the dynamic response of beams with strong quadratic nonlinearity will experience a softening effect while it will show a hardening effect for beams with strong cubic nonlinearities.

**Figure 3:**
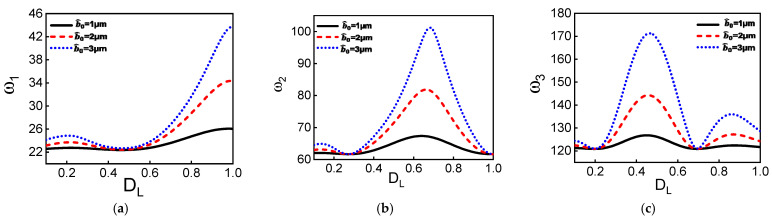

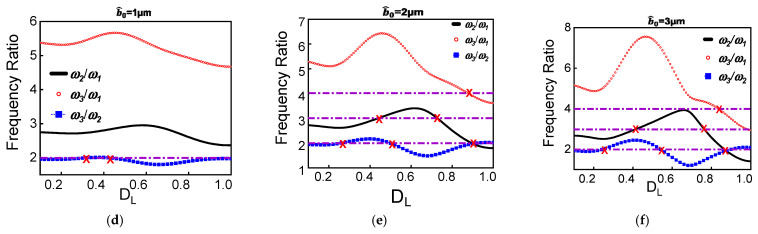
Variation of the three lowest natural frequencies of the proposed hybrid curved beam with dome length. (**a**) The first symmetric natural frequency. (**b**) The first antisymmetric natural frequency. (**c**) The second symmetric natural frequency. (**d**–**f**) Variation of ratios between different modes with the dome length for various initial rises. (**d**) ${\hat{b}}_{0}$= 1 µm. (**e**) ${\hat{b}}_{0}$ = 2 µm. (**f**) ${\hat{b}}_{0}$ = 3 µm. As tuning the dome length, different commensurate ratios between modes could be achieved. The above is a necessary condition for activating the nonlinear coupling between different modes via internal resonance. The *D_L_* axis starts from 0.1. The red crosses are associate with the dome length at which we have commensurate ratios.

Next, we investigate the influence of the dome length on the first three modes of the resonator. As shown in Figure 3a–c, the frequencies vary nonlinearly with the dome length. Also, one can note that for particular dome length values, the initial rise does not affect the frequency values; for example, at *D_L_* around 0.5, the first mode remains constant for different initial rise values. The same can be noted for the second mode at *D_L_* around 0.3 and 1 and the third mode at *D_L_* around 0.25 and 0.7. These results suggest that the geometry parameters have different effects on the effective modal stiffness, allowing a wide range of tunability.

The ratios between different vibration modes are depicted in Figure 3d–f for different initial rises of the dome. Different commensurate ratios (2, 3..., 7) can be obtained between different modes as tuning the dome length. A commensurate ratio between two modes is necessary to activate the nonlinear energy exchange among the involved modes via internal resonance. Figure 3e,f show that for a particular dome length, the ratios between the three lowest frequencies are commensurate (*ω_3_/ω_2_ = ω_2_/ω_1_ = 2*), leading to a potential combined internal resonance and more complex nonlinear energy exchange between involved modes. 

**Figure 4:**
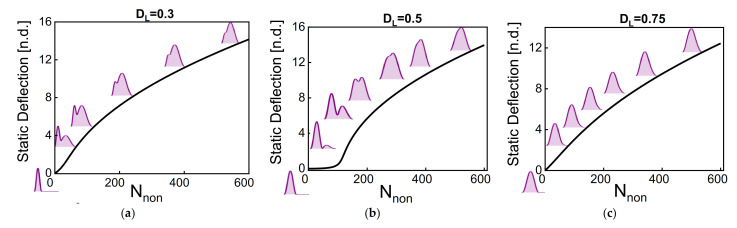

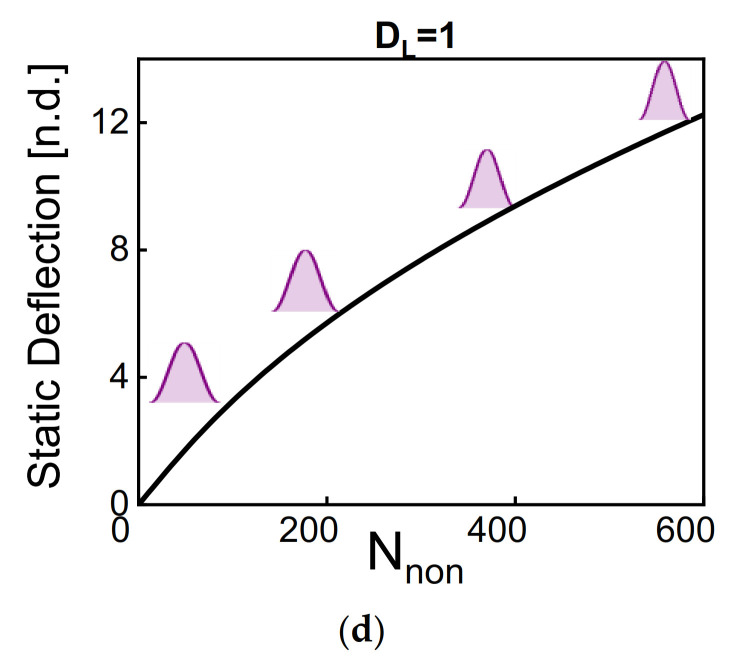
The static deflection with axial load for hybrid shape for different dome lengths. (**a**) *D_L_* = 0.3, (**b**) *D_L_* = 0.5, (**c**) *D_L_* = 0.75, and (**d**) *D_L_* = 1. The initial rise of the dome is equal to 2 µm. The asymmetry of the static shape is clear for lower dome length.

The initial static profile is another parameter of great interest that affects the response dynamics. Here, we study the effect of the axial stress and the dome length on the static deflection by solving Equation (8). For this analysis, the case of ${\hat{b}}_{0}$ = 2 µm is chosen. Figure 4 shows that the dome length significantly influences the hybrid resonator’s static shape as tuning its axial load. For dome length equal to half beam length, the asymmetry is apparent by looking at the displacement at the midpoint, the quarter (the dome-side), and the third-quarter (the straight-side) of the beam. For dome lengths lower than half of the beam length, the static shapes combine a buckled shape on the straight side and the arch shapes on the other side. As the dome length exceeds the half beam length, the static deflection trend is similar to the normal arch. The static profile is important in determining the symmetry and asymmetry of the system (symmetry with respect to the midpoint plane). For asymmetric shapes, the classical crossing between symmetric and antisymmetric modes will be affected and changed to avoid-crossing between both modes.

**Figure 5:**
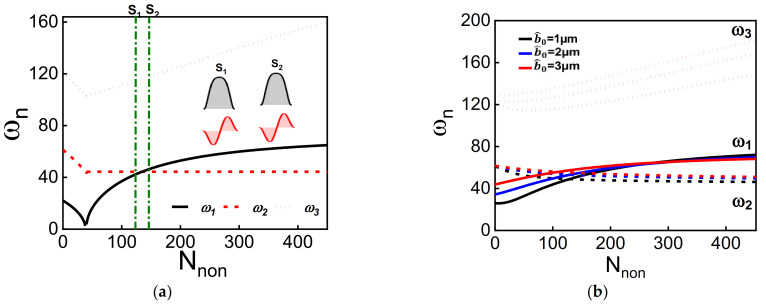
(**a**,**b**): The variation of the three lowest natural frequencies with axial load. (**a**) Case of the straight beam. (**b**) Case of an arch beam with different initial rises. The insets of (**a**) are the associated modes shapes of the first two lowest frequencies before the crossing (S_1_) and after the crossing (S_2_).

**Figure 6:**
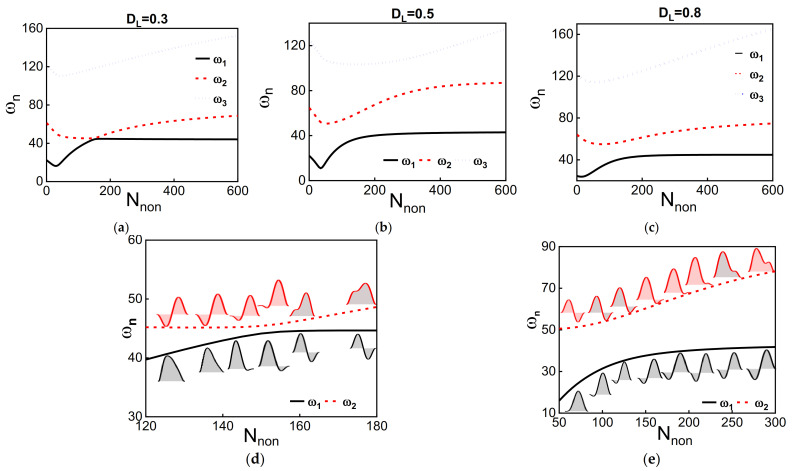
(**a**–**c**): The variation of the three lowest natural frequencies with axial load for different dome lengths, *D_L_ =* 0.3, *D_L_ =* 0.5, and *D_L_ =* 0.8, respectively. The initial rise of the dome is equal to 1 µm. (**d**,**e**) show a zoomed view of the avoided crossing zone between the first two lowest frequencies for *D_L_* = 0.3 and *D_L_* = 0.5, respectively. The insets of (**d**,**e**) are the associated modes shapes of the first two lowest frequencies at different axial load values.

**Figure 7:**
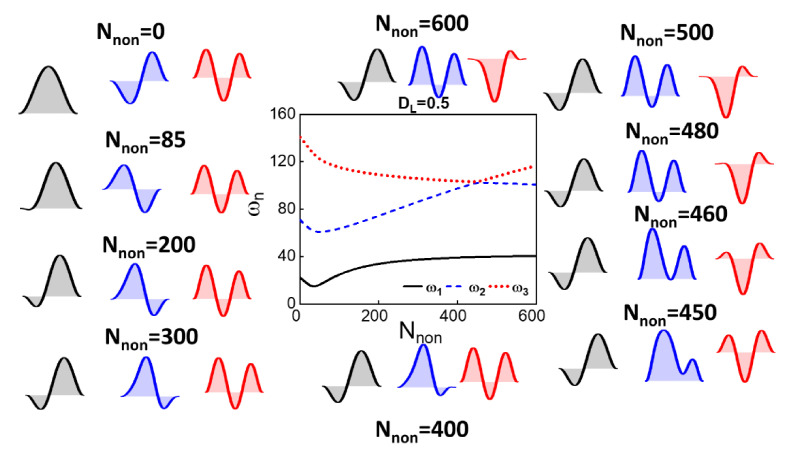

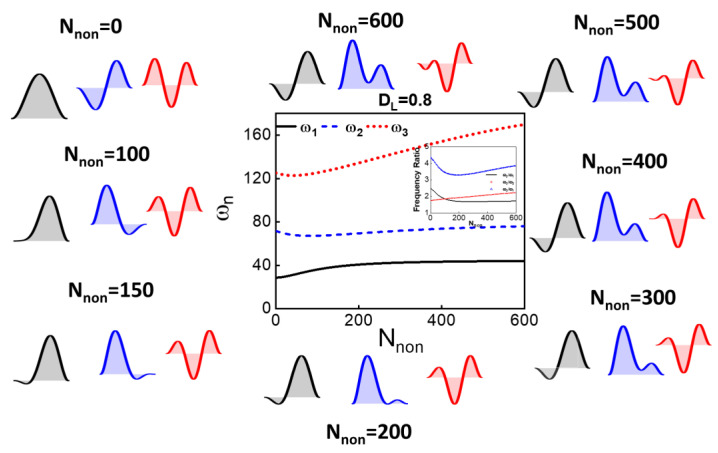
(**a**,**b**): The variation of the three lowest natural frequencies with axial load for different dome lengths, *D_L_* = 0.5 and *D_L_* = 0.8, respectively. The initial rise of the dome is equal to 2 µm. (**a**,**b**) shows the mode shapes associated with the three lowest natural frequencies at different axial loads.

**Figure 8:**
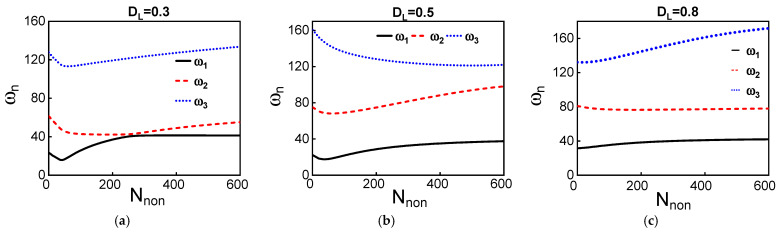
(**a**–**c**): The variation of the three lowest natural frequencies with axial load for different dome lengths, *D_L_* = 0.3, *D_L_* = 0.5, and *D_L_* = 0.8, respectively. The initial rise of the dome is equal to 3 µm.

Another parameter that we explore its effect on the frequency values is the axial stress (compressive load). The axial load could be controlled by different transduction mechanisms, such as the electrothermal voltage [22,23] and sided electrostatic electrode [41]. Beams with tunable axial load show good capabilities in several potential applications, mainly thermal-conductivity-based gas and pressure sensors, force sensors, logic memories, and temperature sensors [7,50]. Figure 5, Figure 6, Figure 7 and Figure 8 show the variation of the lowest three natural frequencies as tuning the axial load (compressive load) for various dome lengths and initial rises. Figure 5 shows the variation of the lowest three frequencies with axial load for the classical cases: straight beam (Figure 5a) and standard arch beam (Figure 5b). As classically known, the frequencies of straight beam decrease at higher axial load values until reaching buckling. After buckling, the frequencies of the first and second symmetric modes increase with axial load, while the first antisymmetric mode remains constant. This leads to a crossing between the first symmetric and the first antisymmetric mode. As shown in the insets of Figure 5a, both modes do not interact with each other at the crossing zone; hence no mode hybridization is reported. The same phenomenon is reported for the arch beam case when the first symmetric and antisymmetric modes cross. One should note that the buckling instability disappears when introducing an initial curvature to the structure, Figure 5b. Also, the antisymmetric modes for the initially curved structures decrease with axial loads for the whole range of compressive axial loads contrary to the straight beam configuration. 

**Figure A1:**
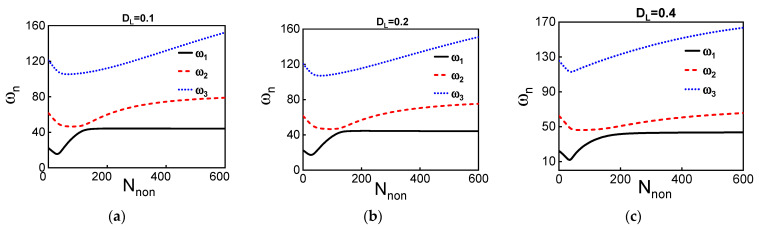

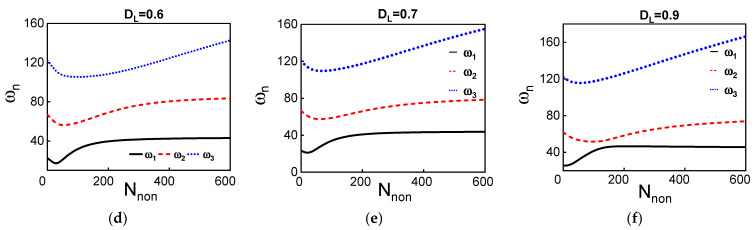
(**a**–**f**): The variation of the three lowest natural frequencies with axial load for different dome lengths. The initial rise of the dome is equal to 1 µm.

Next, we investigate the effect of the dome length and axial load on the frequency values of the first three modes for initial rise *b_0_* = 1 µm, as depicted in Figure 6 and Figure A1. The results show that the first two modes are more sensitive to the dome length compared with the third mode. The third mode seems to have the same qualitative behavior as increasing the axial load without interaction with the other modes. The two lowest frequencies initially decrease with axial load, then increase after reaching a threshold axial load value. These two frequencies should cross without interacting at a certain axial load for a normal arch or buckled beam, as shown in Figure 5. However, the presence of the dome creates an asymmetry that transforms the crossing into avoided crossing between both modes. The avoided crossing frequency width varies for different dome lengths.

In contrast to the arch beam or buckled beam, the first two lowest modes hybridize around the avoid-crossing zone. The hybridization of both first symmetric and antisymmetric modes leads to the same out-of-phase mode shapes at the avoid-crossing zone. The above is similar to the case of an arch beam under asymmetric electrostatic actuation [44]. The first two lowest modes show an avoided crossing for low values of dome lengths that are smaller than 0.5 *L_hb_*. The inset of Figure 6d shows the mode shapes at different axial load values around the avoided-crossing region for dome length *D_L_* = 0.3; the two modes hybridize, and then interchange mode shapes similar to the veering phenomena. For dome lengths equal to 0.5 *L_hb_*, Figure 6e, the two modes show a similar behavior but with a higher frequency difference between them. Even if the frequency difference is high, an alteration of mode shapes is reported for this case, as seen in Figure 6e. Hence, the presence of the dome creates an asymmetry in the beam that leads to the veering between different modes as tuning their stiffness without crossing.

**Figure A2:**
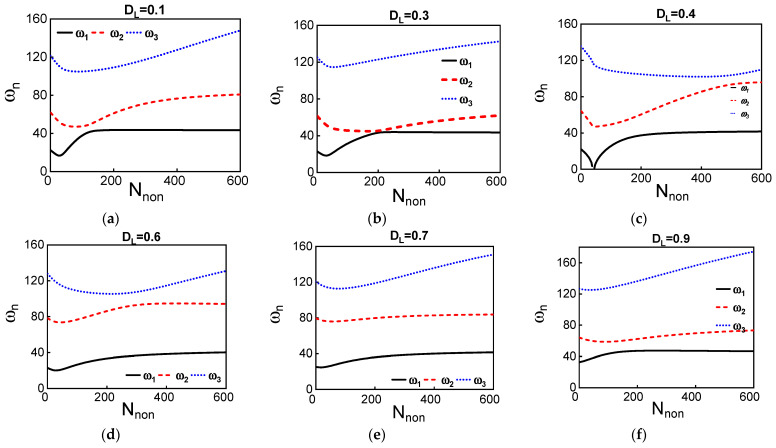
(**a**–**f**): The variation of the three lowest natural frequencies with axial load for different dome lengths. The initial rise of the dome is equal to 2 µm.

For initial rise $\hat{b}$*_0_* = 2 µm, the frequency tuning shows more mode coupling as varying the dome length and the axial load, as shown in Figure 7 and Figure A2. Figure 7a shows, for the case of *D_L_* = 0.5, the ability to have veering between the second and third modes and crossing between the first and second modes. As shown in the insets of Figure 7a, the mode shapes show the hybridization of the two modes around the veering zone. The interchange between the two lowest modes is also demonstrated for lower axial load even though the difference between the two frequencies is high. Another feature that could be seen for dome length *D_L_* = 0.8, the two lowest frequencies vary with an almost constant ratio for a wide range of axial load, as shown in Figure 7b. The results show the potential of utilizing the proposed resonators as stable resonators in a harsh environment or for temperature compensation through internal resonance. The energy transfer among the coupled modes acts as a stabilizing feedback loop that suppresses the amplitude and frequency fluctuations [10].

As tuning the geometric parameters and the dome length, we showed that the type and magnitude of nonlinearity could be controlled. The ratio can also be tuned to a commensurate number. The above would be necessary to activate the nonlinear coupling between the modes via internal resonance for a wide range of axial load [46]. Operating a resonator in the internal resonance zone increases the resonator’s frequency stability due to nonlinear energy transfer between the two involved modes [10,51]. The proposed system shows the potential to be operated in an internal resonance zone for a wide range of axial loads values, which mimics temperature fluctuation. 

**Figure A3:**
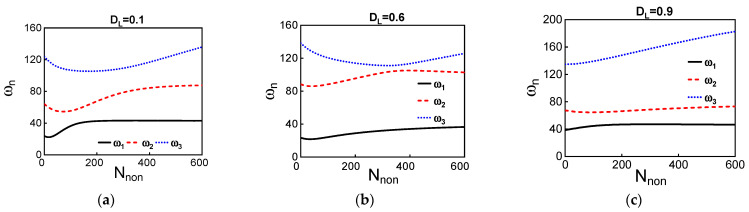
(**a**–**c**): The variation of the three lowest natural frequencies with axial load for different dome lengths. The initial rise of the dome is equal to 3 µm.

Increasing the initial rise to $\hat{b}$*_0_* = 3 µm will change the coupling behavior among modes for different dome lengths. However, the same features could be seen such as veering (avoid-crossing) and constant frequency ratios, as depicted in Figure 8 and Figure A3. Different frequency tuning and coupling phenomena could be demonstrated by carefully choosing the initial rise and the dome length. This depends on the targeted applications. The presented approach here is scalable, so some features could be achieved for higher or lower frequency ranges by enlarging or shrinking the beam sizes. 

**Figure 9:**
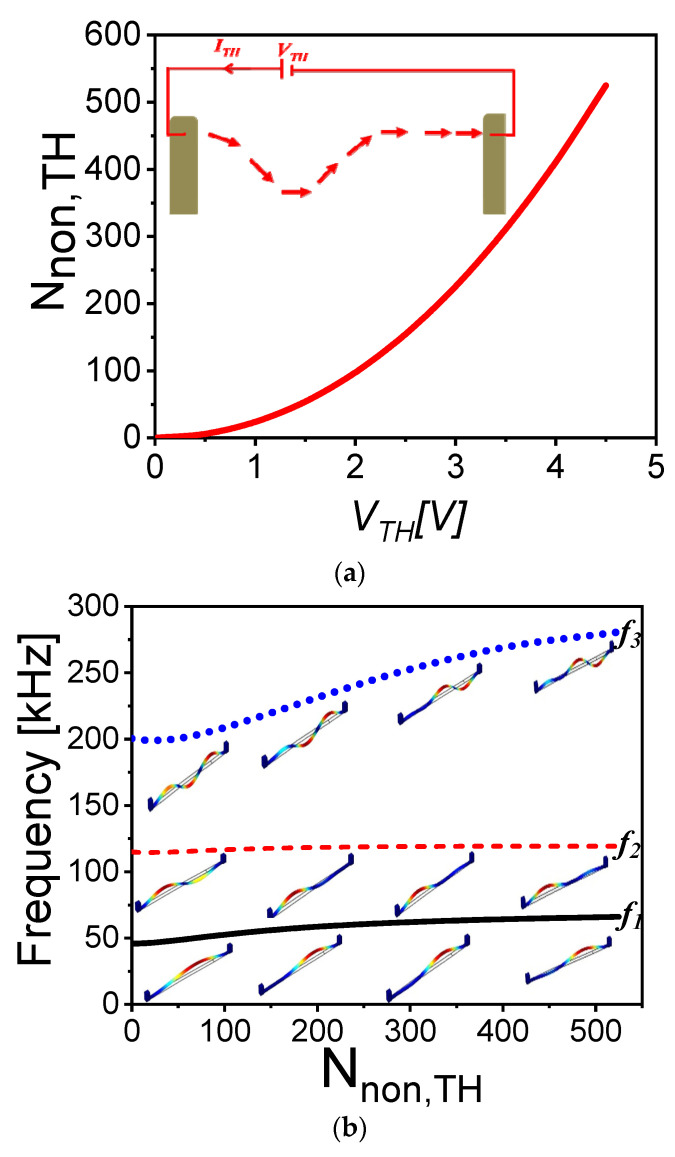
(**a**) Variation of the nondimensional axial stress with the applied electrothermal voltage. The inset in (**a**) presents the schematic of the electrothermally tuned hybrid resonator. (**b**) The variation of the three lowest natural frequencies with nondimensional axial load for dome length equal to 0.8 *L_hb_* and initial rise equal to 2 µm. The insets in (**b**) present the mode shapes of the corresponding frequencies at different axial load (i.e., different *V_TH_*).

Next, a 3D multi-physics finite-element model, using the commercial finite element software COMSOL [51], is conducted to validate the numerical model presented in this work and verify that the various assumptions made in the analytical model are valid. The hybrid beam is simulated using the parameters given in Table 1. The initial rise and dome length are equal to 2 µm and 0.8 *L_hb_*, respectively. In order to simulate the effect of axial load, electrothermal actuation was used [23]. Electrothermal tuning of MEMS resonators was deeply investigated in the literature in the case of the bridge [21], arch [22], V-shaped [27], and U-shaped [52] resonators, and were demonstrated for several potential applications, such as filtering, logic memories, and pressure/gas sensing. The Solid Mechanics, Electric Currents, and Heat Transfer interfaces are considered to account for the various physical domains of the problem as described in [23]. One should mention that the selection of the electrothermal tuning is based on its popularity and simplicity of this method in tuning the axial stress of microresonators. The relation between the nondimensional induced stress ($N_{non,TH}=\frac{L_{hb}^{2}}{EI}{\hat{S}}_{TH}$) and the electrothermal voltage is plotted in Figure 9a, where ${\hat{S}}_{TH}$ is the thermal induced axial stress extracted from COMSOL. Figure 9b reveals the variation of the three lowest natural frequencies of the hybrid resonator with the nondimensional axial load (induced by the corresponding electrothermal voltage) showing a good agreement with the numerical data presented in Figure 7b.

## 4. Discussion

This study proposes a new hybrid structure combining the straight and arch beam shapes. The proposed design is simple in principle and easy to fabricate using the standard two-mask microfabrication process of silicon-on-insulator (SOI) wafers, more details can be found in [17]. A passive and active systematic methodology is demonstrated to tailor the system nonlinearities by controlling the initial shaped and geometric parameters. The results demonstrate the possibility of realizing structures with strong cubic, quadratic, or even zero effective nonlinearity. The proposed design shows different ways of tuning the frequencies by adequately selecting the dome length and initial rise values. Avoid-crossing and mode-hybridization between different modes are demonstrated for various geometric parameters. Depending on the targeted applications and performance metrics, certain geometric parameters can be selected. For example, Figure 8c shows the possibility of designing a structure with a stable second mode frequency over a wide range of axial stress. This characteristic is vital for timing and sensing devices operated in unstable temperature conditions. Also, we show the possibility of achieving commensurate ratios necessary for activating nonlinear energy transfer between the different modes. Figure 3d shows the possibility of realizing a structure with a 2:1 frequency ratio over a wide range of dome lengths, which allows large tolerance in the fabrication process. Moreover, our results show the possibility of realizing a structure with a 2:1 frequency ratio over a wide range of axial stress, inset of Figure 7b, such structure has a great potential in energy harvesting applications operated in unstable temperature conditions. 

One should note that more studies need to be done to characterize the nonlinear dynamics of the proposed device under different actuation forces and different axial load configurations. Driving the proposed designs using electrostatic force is expected to have rich and complex linear and nonlinear dynamics phenomena; Snap-through, pull-in bifurcations, switching between softening and hardening. Also, using partial electrodes at different positions with respect to the dome length will influence the device static and dynamic behavior. These complex and rich dynamics can be exploited to enhance the performance of the resonators or avoid it for safe implementation of the resonator. 

## Figures and Tables

**Table 1:** Geometrical and mechanical properties of the silicon hybrid MEMS resonator.

Quantity	Values
Length, *L_hb_*	700 (μm)
Thickness, *h_hb_*	2 (μm)
Width, *b_hb_*	25 (μm)
Young’s Modulus, *E*	169 (Gpa)
Density, ρ	2332 (kg·m^−3^)

## Appendix A

In order to extract the nonlinear cubic and quadratic coefficients of the system, we referred to [44], where a detailed multiple scaled derivation was presented for initially curved structures. The nonlinear coefficients are computed at zero electrostatic force and zero axial load for various initial shapes. We rewrite the main equations linked to the solved problem.

For simplicity, we introduce

(A1) $$\Gamma \left(f_{1}\left(x,t\right),f_{2}\left(x,t\right)\right)=\int_{0}^{1}\frac{\partial f_{1}}{\partial x}\frac{\partial f_{2}}{\partial x}dx$$

(A2) $$\langle f_{1},f_{2}\rangle =\int_{0}^{1}f_{1}\left(x\right)\;f_{2}\left(x\right)\;dx$$

(A3) $$S=S_{q}+S_{c}$$

*S*, *Sq*, *Sc*, denote the geometric nonlinear coefficient, its quadratic order, and its cubic order. *Sq* and *Sc* are defined as below:

(A4) $$S_{q}=\int_{0}^{1}\chi_{q}\;\varphi_{1}\;dx$$

(A5) $$S_{c}=\int_{0}^{1}\chi_{c}\;\varphi_{1}\;dx$$

The functions *χ_q_* and *χ_c_* are defined as:

(A6) $$\chi_{q}=Г\left(\varphi_{1},\psi \right)\langle w_{hb,0}^{\prime \prime },\varphi_{1}\rangle +Г\left(w_{hb,0},\varphi_{1}\right)\langle {\varphi_{1}}^{\prime \prime },\varphi_{1}\rangle +Г\left(w_{hb,0},\varphi_{1}\right)\langle \psi^{\prime \prime },\varphi_{1}\rangle$$

(A7) $$\chi_{c}=\frac{3}{2}Г\left(\varphi_{1},\varphi_{1}\right)\langle {\varphi_{1}}^{\prime \prime },\varphi_{1}\rangle$$

The function ψ is obtained by solving the boundary value problem given by

(A8) $$\psi \left(x\right):H\left[0\right]=\Lambda$$

where H and Λ are defined by

(A9) $$H\left[\omega_{1}\right]=H^{\left(4\right)}-N_{0}\;H^{\prime \prime }-w_{\mathrm{hb},0}{}^{\prime \prime }\left(\int_{0}^{1}w_{\mathrm{hb},0}{}^{\prime }H^{\prime }dx\right)-\omega_{1}{}^{2}H$$

(A10) $$\Lambda =\frac{1}{2}w_{\mathrm{hb},0}{}^{\prime \prime }\Gamma \left(\varphi_{1},\varphi_{1}\right)+\varphi_{1}{}^{\prime \prime }\Gamma \left(w_{\mathrm{hb},0},\varphi_{1}\right)$$

## Appendix B

The variation of the three lowest natural frequencies with axial load for different dome length *D_L_* and for different initial rises ${\hat{b}}_{0}$.

## References

[1] Yamagiwa H., Sato S., Fukawa T., Ikehara T., Maeda R., Mihara T., Kimura M. (2014). Detection of Volatile Organic Compounds by Weight-Detectable Sensors coated with Metal-Organic Frameworks. Sci. Rep..

[2] Vyas A., Peroulis D., Bajaj A.K. (2009). A Microresonator Design Based on Nonlinear 1 : 2 Internal Resonance in Flexural Structural Modes. J. Microelectromech. Syst..

[3] Zhang T., Wei X., Jiang Z., Cui T. (2018). Sensitivity enhancement of a resonant mass sensor based on internal resonance. Appl. Phys. Lett..

[4] Bannon F.D., Clark J.R., Nguyen C.-C. (2000). High-Q HF microelectromechanical filters. IEEE J. Solid-State Circuits.

[5] Pallay M., Miles R.N., Towfighian S. (2021). Towards a high bias voltage MEMS filter using electrostatic levitation. Mech. Syst. Signal Process..

[6] Mahboob I., Flurin E., Nishiguchi K., Fujiwara A., Yamaguchi H. (2011). Interconnect-free parallel logic circuits in a single mechanical resonator. Nat. Commun..

[7] Al Hafiz M.A., Kosuru L., Younis M.I. (2016). Microelectromechanical reprogrammable logic device. Nat. Commun..

[8] Yang W., Towfighian S. (2017). Internal resonance and low frequency vibration energy harvesting. Smart Mater. Struct..

[9] Du S., Jia Y., Zhao C., Amaratunga G.A.J., Seshia A.A. (2019). A fully integrated split-electrode SSHC rectifier for piezoelectric energy harvesting. IEEE J. Solid-State Circuits.

[10] Antonio D., Zanette D.H., López D. (2012). Frequency stabilization in nonlinear micromechanical oscillators. Nat. Commun..

[11] Nguyen C.T.-C. (2007). MEMS technology for timing and frequency control. IEEE Trans. Ultrason. Ferroelectr. Freq. Control.

[12] Lyshevski S.E. (2018). MEMS and NEMS: Systems, Devices, and Structures.

[13] Liu Y., Yang J., Liu Z., Cheng Y., Grey F., Zheng Q. (2013). Mechanics and Multidisciplinary Study for Creating Graphene-Based van der Waals Nano/Microscale Devices. IUTAM Symposium on Surface Effects in the Mechanics of Nanomaterials and Heterostructures.

[14] Potekin R., Dharmasena S., Keum H., Jiang X., Lee J., Kim S., Bergman L.A., Vakakis A.F., Cho H. (2018). Multi-frequency Atomic Force Microscopy based on enhanced internal resonance of an inner-paddled cantilever. Sens. Actuators A Phys..

[15] Westra H.J.R., van der Zant H.S.J., Venstra W.J. (2012). Modal interactions of flexural and torsional vibrations in a microcantilever. Ultramicroscopy.

[16] Jeong B., Pettit C., Dharmasena S., Keum H., Lee J., Kim J., Kim S., McFarland D.M., Bergman L.A., Vakakis A.F. (2016). Utilizing intentional internal resonance to achieve multi-harmonic atomic force microscopy. Nanotechnology.

[17] Nabavi S., Zhang L. (2019). Nonlinear multi-mode wideband piezoelectric mems vibration energy harvester. IEEE Sens. J..

[18] Hajjaj A.Z., Hafiz M.A., Younis M.I. (2017). Mode Coupling and Nonlinear Resonances of MEMS Arch Resonators for Bandpass Filters. Sci. Rep..

[19] Qalandar K.R., Strachan B.S., Gibson B., Sharma M., Ma A., Shaw S.W., Turner K.L. (2014). Frequency division using a micromechanical resonance cascade. Appl. Phys. Lett..

[20] Kozinsky I., Postma H.W.C., Bargatin I., Roukes M.L. (2006). Tuning nonlinearity, dynamic range, and frequency of nanomechanical resonators. Appl. Phys. Lett..

[21] Kacem N., Hentz S. (2009). Bifurcation topology tuning of a mixed behavior in nonlinear micromechanical resonators. Appl. Phys. Lett..

[22] Hajjaj A.Z., Alcheikh N., Ramini A., Al Hafiz M.A., Younis M.I. (2016). Highly Tunable Electrothermally and Electrostatically Actuated Resonators. J. Microelectromech. Syst..

[23] Hajjaj A.Z., Alcheikh N., Younis M.I. (2017). The static and dynamic behavior of MEMS arch resonators near veering and the impact of initial shapes. Int. J. Non-Linear Mech..

[24] Zhang G., Zhao L., Xu L., Jiang Z., Zhao Y., Wang X., Liu Z. (2013). Active Frequency Tuning for Magnetically Actuated and Piezoresistively Sensed MEMS Resonators. IEEE Electron. Dev. Lett..

[25] Cho H., Jeong B., Yu M.-F., Vakakis A.F., McFarland D.M., Bergman L.A. (2012). Nonlinear hardening and softening resonances in micromechanical cantilever-nanotube systems originated from nanoscale geometric nonlinearities. Int. J. Solids Struct..

[26] Alcheikh N., Ouakad H.M., Younis M.I. (2021). Dynamic analysis of straight stepped microbeams. Int. J. Non-Linear Mech..

[27] Alcheikh N., Ouakad H.M., Younis M.I. (2020). Dynamics of V-Shaped Electrothermal MEMS-Based Resonators. J. Microelectromech. Syst..

[28] Dou S., Strachan B.S., Shaw S.W., Jensen J.S. (2015). Structural optimization for nonlinear dynamic response. Philos. Trans. R. Soc. A Math. Phys. Eng. Sci..

[29] Li L.L., Polunin P.M., Dou S., Shoshani O., Scott Strachan B., Jensen J.S., Shaw S.W., Turner K.L. (2017). Tailoring the nonlinear response of MEMS resonators using shape optimization. Appl. Phys. Lett..

[30] Tripathi A., Bajaj A.K. (2016). Topology optimization and internal resonances in transverse vibrations of hyperelastic plates. Int. J. Solids Struct..

[31] Shoshani O., Dykman M.I., Shaw S.W. (2020). Tuning linear and nonlinear characteristics of a resonator via nonlinear interaction with a secondary resonator. Nonlinear Dyn..

[32] Tamayo J., Kosaka P.M., Ruz J.J., San Paulo Á., Calleja M. (2013). Biosensors based on nanomechanical systems. Chem. Soc. Rev..

[33] Raman A., Melcher J., Tung R. (2008). Cantilever dynamics in atomic force microscopy. Nano Today.

[34] Lifshitz R., Cross M.C. (2008). Nonlinear dynamics of nanomechanical and micromechanical resonators. Rev. Nonlinear Dyn. Complex..

[35] Asadi K., Yu J., Cho H. (2018). Nonlinear couplings and energy transfers in micro-and nano-mechanical resonators: Intermodal coupling, internal resonance and synchronization. Philos. Trans. R. Soc. A Math. Phys. Eng. Sci..

[36] Hajjaj A.Z., Jaber N., Ilyas S., Alfosail F.K., Younis M.I. (2019). Linear and nonlinear dynamics of micro and nano-resonators: Review of recent advances. Int. J. Non-Linear Mech..

[37] Nitzan S.H., Zega V., Li M., Ahn C.H., Corigliano A., Kenny T.W., Horsley D.A. (2015). Self-induced parametric amplification arising from nonlinear elastic coupling in a micromechanical resonating disk gyroscope. Sci. Rep..

[38] Zhou X., Zhao C., Xiao D., Sun J., Sobreviela G., Gerrard D.D., Chen Y., Flader I., Kenny T.W., Wu X. (2019). Dynamic modulation of modal coupling in microelectromechanical gyroscopic ring resonators. Nat. Commun..

[39] Alneamy A.M., Khater M.E., Abdel-Aziz A.K., Heppler G.R., Abdel-Rahman E.M. (2020). Electrostatic arch micro-tweezers. Int. J. Non-Linear Mech..

[40] Tajaddodianfar F., Pishkenari H.N., Yazdi M.R.H., Miandoab E.M. (2015). Size-dependent bistability of an electrostatically actuated arch NEMS based on strain gradient theory. J. Phys. D Appl. Phys..

[41] Alcheikh N., Ramini A., Hafiz MAAl Younis M.I. (2017). Tunable clamped–guided arch resonators using electrostatically induced axial loads. Micromachines.

[42] Wang D.F., Chatani K., Ikehara T., Maeda R. (2012). Mode localization analysis and characterization in a 5-beam array of coupled nearly identical micromechanical resonators for ultra-sensitive mass detection and analyte identification. Microsyst. Technol..

[43] Erbes A., Thiruvenkatanathan P., Woodhouse J., Seshia A.A. (2014). Numerical study of the impact of vibration localization on the motional resistance of weakly coupled MEMS resonators. J. Microelectromech. Syst..

[44] Hajjaj A.Z., Alfosail F.K., Jaber N., Ilyas S., Younis M.I. (2019). Theoretical and experimental investigations of the crossover phenomenon in micromachined arch resonator: Part II—simultaneous 1:1 and 2:1 internal resonances. Nonlinear Dyn..

[45] Hajjaj A.Z., Jaber N., Hafiz M.A.A., Ilyas S., Younis M.I. (2018). Multiple internal resonances in MEMS arch resonators. Phys. Lett. A.

[46] Xia C., Wang D.F., Ono T., Itoh T., Maeda R. (2020). A mass multi-warning scheme based on one-to-three internal resonance. Mech. Syst. Signal Process..

[47] Hacker E., Gottlieb O. (2012). Internal resonance based sensing in non-contact atomic force microscopy. Appl. Phys. Lett..

[48] Pu D., Wei X., Xu L., Jiang Z., Huan R. (2018). Synchronization of electrically coupled micromechanical oscillators with a frequency ratio of 3: 1. Appl. Phys. Lett..

[49] Ouakad H.M., Younis M.I. (2010). The dynamic behavior of MEMS arch resonators actuated electrically. Int. J. Non-Linear Mech..

[50] Hajjaj A.Z., Jaber N., Alcheikh N., Younis M.I. (2019). A Resonant Gas Sensor Based on Multimode Excitation of a Buckled Microbeam. IEEE Sens. J..

[51] COMSOL. https://www.comsol.com/.

[52] Qiu J., Lang J.H., Slocum A.H., Weber A.C. (2005). A bulk-micromachined bistable relay with U-shaped thermal actuators. J. Microelectromech. Syst..

